# Waist-to-Hip Ratio, but Not Body Mass Index, Is Associated with Testosterone and Estradiol Concentrations in Young Women

**DOI:** 10.1155/2015/654046

**Published:** 2015-08-17

**Authors:** Ricardo Mondragón-Ceballos, Mónica Dafne García Granados, Ana Lilia Cerda-Molina, Roberto Chavira-Ramírez, Leonor Estela Hernández-López

**Affiliations:** ^1^Departamento de Etología, Instituto Nacional de Psiquiatría, “Ramón de la Fuente Muñiz”, Calzada México-Xochimilco 101, Colonia San Lorenzo Huipulco, Tlalpan, 14370 México, DF, Mexico; ^2^Departamento de Biología de la Reproducción, Instituto Nacional de Ciencias Médicas y Nutrición Salvador Zubirán, Calle Vasco de Quiroga 15, Colonia Sección XVI, Tlalpan, 14000 Mexico, DF, Mexico

## Abstract

We studied if testosterone and estradiol concentrations are associated with specific female waist-to-hip ratios (WHRs) and body mass indices (BMIs). Participants were 187 young women from which waist, hips, weight, and height were measured. In addition, participants informed on which day of their menstrual cycle they were and provided a 6 mL saliva sample. Ninety-one of them were in the follicular phase and 96 in the luteal phase. Only in the fertile phase of the menstrual cycle we found a significant interaction between testosterone and estradiol affecting WHR (*b* ± s.e. = −0.000003 ± 0.000001;  *t*_94_ = −2.12, adjusted *R* ^2^ = −0.008,  *P* = 0.03). Women with the highest levels of both hormones had the lowest WHRs, while women with low estradiol and high testosterone showed the highest WHRs. BMI significantly increased as testosterone increased in female in their nonfertile days.

## 1. Introduction

Given the current worldwide obesity crisis, waist-to-hip ratio (WHR) and body mass index (BMI) are recommended anthropometric measures to physicians to serve as proxies of metabolic and cardiovascular risk in women and men, with WHR being a better predictor of cardiovascular disease and diabetes than BMI ([1–3], but see [4]). It is known that as WHR and BMI increase, there is a higher risk of incurring into a variety of diseases, ranging from hypertension to diabetes, cancer, fertility problems, and depression [1, 4–6].

From a different perspective, evolutionary psychology, knowing that a low female WHR is associated with increased fecundity, Singh [7] showed that men rate female with low WHRs, particularly those equal or close to 0.7, as the most attractive, without BMI having such a strong effect. Low WHRs are attractive to men [7–12], even cross-culturally [13–15]. However, mean WHR differs between human populations; women from non-Western countries have on average higher WHR than women from Western countries [16]. Women with high WHRs have more sons than those with a lower WHR, which in turn have more daughters [17]. Moreover, besides cardiovascular risk, women with high WHRs suffer from anxiety, low self-esteem, and anger and receive less social support [18].

High estradiol concentrations are deemed as responsible for maintaining a low WHR, since this steroid regulates fat accumulation in the buttocks, hip, thighs, and bosom [19, 20]. Estradiol also affects women behavior and personality. Women with low estrone-3-glucuronide (the main metabolite of estradiol) levels exhibit stronger ovulatory shifts in preferences for masculine male voices during the late follicular phase and feminine male voices during the luteal phase than do women with high estrone-3-glucuronide levels [21]. During the late follicular phase, the reported ideal number of children increases with estrone-3-glucuronide levels in young nulliparous women [22], and ratings of the attractiveness of photographs of male faces during this phase are positively correlated with women's estradiol concentrations and the testosterone concentrations of the pictured men [23]. Overbidding (i.e., bidding that exceeds the risk-neutral Nash equilibrium bid) in auction games increases along with estradiol levels in women [24]. Implicit power motivation (a concern for having an impact on others or the world at large) is positively correlated with estradiol levels, and affiliation motivation (a concern for establishing, maintaining, or restoring close, friendly relationships) is high at midcycle [25–27]. High estradiol concentrations are associated with low levels of attachment avoidance and high levels of intimacy motivation, two personality traits involved in looking for and maintaining emotional intimacy [28]. On the other hand, besides high avoidance attachment and high attachment anxiety, women with low basal estradiol levels show an attenuated estradiol response to viewing emotional, nonsexual, and intimacy scenes, such as daughter-father interactions [29]. Concerning female sexuality, estradiol is positively correlated with overall sexual desire and the Sensory Dimension [30] of the Orgasm Checklist [31]. Weak sexual arousal and unsatisfactory orgasmic experiences are common in women with low estradiol concentrations [30]. Women having high estradiol concentrations throughout the late follicular phase experience more jealous feelings to imagined partner's sexual infidelity and more anger than hurt to imagined partner's emotional infidelity [32, 33].

Recent studies have revealed an outstanding role of feminine testosterone in metabolic and psychological processes, including WHR. The action of testosterone on WHR seems contrary to that of estradiol. High WHRs are correlated with high testosterone levels in pre- and perimenopausal women [34–36] or in medical conditions where this hormone is naturally augmented such as in polycystic syndrome or morbid obesity [36, 37]. Concerning sexuality, women with high androgen levels experience more orgasms [38], report more satisfactory orgasmic experiences [39], have greater well-being [40], tend to be promiscuous [41], and exhibit increased attraction to masculine faces in the late follicular days [42]. Socially, they are dominant persons [43], are usually involved in long-term relationships [44], and achieve greater occupational reputation [45]. However, they score low on the “children lover” and “maternal” items of the Bem Sex Role Inventory (BSRI) [46]. As entrepreneurs they exhibit less risk aversion in auction games [24], exhibit increased risk seeking for losses, and are more verbally aggressive [16].

We studied the relation between salivary estradiol and testosterone with WHR and BMI in a population of young healthy women. The main idea was to search for a role of these steroids in eliciting interindividual variation in these two anthropometric measures. In particular, we were interested in WHR, which has been correctly identified as an “honest” signal of potential female fertility, supported by gynecological findings (see references above). Considering that in women estrogens promote fat accumulation in hips, buttocks, thighs, and bosom [19, 20], while androgens promote fat accumulation in the abdomen, leading to weight increase [34, 35], we expected that the lowest WHRs would be associated with high levels of estradiol, while both high WHRs and BMIs would be positively correlated with testosterone.

## 2. Material and Methods

### 2.1. Subjects

One hundred eighty-seven women voluntarily participated in the present work. Volunteers were students recruited in various faculties at the Universidad Nacional Autónoma de México and the Escuela Nacional de Antropología e Historia in Mexico City. To be eligible, participants were required to be either in the second week of the menstrual phase (late follicular) or in the third to fourth week (luteal phase). All participants signed an agreement stating they had read and understood they were taking part in a nonintrusive research protocol and have no impediment to do so. For taking part in the study, participants received a monetary compensation (~$20.00 USD at the time of the study). Participants were not asked to provide written name or any other confidential data but had to agree on being measured and give age. In addition, participants were required not to be using hormonal or chemical contraceptives, to have had regular menstrual cycles (28–30 days) during the last 6 months, and not to suffer from any sort of gynecological illness.  Table 1 shows the characteristics of our sample of participants.

### 2.2. Ethical Note

The Bioethics Committee of the Instituto Nacional de Psiquiatría “Ramón de la Fuente Muñiz” approved our research protocol. The study conforms to the guidelines of the Mexican Official Norm and the World Health Organization regulations for research with human beings.

### 2.3. Procedure

Participants were asked to present at the lab between 0900 and 1100 h when testosterone levels are high [47, 48], to be measured and provide a 6 mL saliva sample (collected in sterile polypropylene vials) for hormonal assessment, and inform if they had or not passed their fertile days. Height, waist, and hip circumference were obtained using a measurement tape and weight was measured using an electronic floor scale (0.1 kg precision). Waist circumference was measured at the narrowest point between the chest and hips. Hip circumference was measured around the hips and buttocks.

Saliva samples were immediately frozen in acetone and dry ice and stored at −70°C. Afterwards samples were freed from mucopolysaccharides by subjecting them to 4 subsequent freeze-thaw cycles and centrifugation (3000 rpm × 30 min), collecting the supernatants in each cycle to proceed to the next freeze-thaw cycle. We measured testosterone by quimioluminiscence (IMMULITE 1000 Siemens counter, ciudad). The minimum testosterone measured was 20 ng/dL and the maximum 160 ng/dL. Interassay and intra-assay coefficients were 8.95% and 8.02%, respectively.

We used RIA to measure estradiol in duplicate, using solid-phase Coat-A-Count ^125^I RIA. The estradiol standard was diluted 30 times in albumin buffer 4% pH 7.2. Interassay and intra-assay coefficients were 7.84% and 7.01%, respectively.

### 2.4. Analyses

To ensure that participants did know in which phase of the menstrual cycle (late follicular or luteal) they were, comparisons between measurements obtained from women in the fertile (late follicular) and nonfertile (luteal) days were done by means of *t*-tests. To test the relation between estradiol and testosterone and WHR and BMI we used Hierarchical Linear Regression. Independent variables were entered in the next order: (1) estradiol, (2) testosterone, and (3) the testosterone × estradiol interaction. Following Aiken and West [49] method to plot and obtain slopes of interactions in Multiple Regression, testosterone and estradiol mean-centered data were used in the Hierarchical Linear Regressions. Analyses were done using SPSS 17 (SPSS Inc., Chicago, IL, USA). All tests are two-tailed, and significance was set at *P* ≤ 0.05.

## 3. Results

Table 1 shows the descriptive statistics of our sample. Participants in the fertile and the nonfertile phase were on average the same age, although the oldest participant pertains to the nonfertile sample. Our sample included 4 undernourished women (BMI < 18.5), 2 in the fertile phase and 2 in the nonfertile phase; 31 overweight participants (25 < BMI < 30), 2 in the fertile phase and 29 in the nonfertile phase; 15 obese (BMI ≥ 30), 6 in the fertile phase and 9 in the nonfertile. The WHR of women in the nonfertile phase was nonsignificantly higher than those from the fertile phase (*t*_185_ = 1.71, *P* = 0.09), but BMIs were on average equally distributed. Estradiol was significantly higher in the fertile phase (*t*_185_ = 10.4, *P* < 0.0001) and so was testosterone (*t*_185_ = 4.02, *P* < 0.0001).

**Figure 1:**
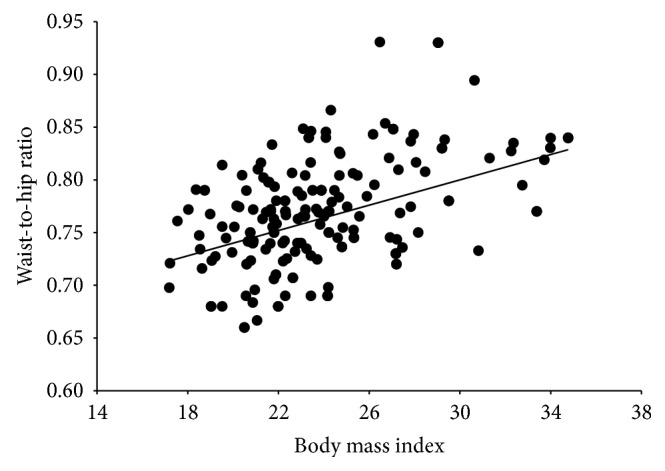
Scatterplot showing the relationship between body mass index and waist-to-hip ratio of female participants in our study. Both variables were significantly correlated (*P* < 0.0001).

Appreciate in Figure 1 that, in the total sample, WHR and BMI had a significant positive correlation (*b* ± s.e. = 0.01 ± 0.001; *t*_185_ = 7.05, *R* ^2^ = 0.21, *P* < 0.0001). This positive association did not change with phase (fertile: *b* ± s.e. = 0.01 ± 0.001; *t*_89_ = 5.8, *R* ^2^ = 0.27, *P* < 0.0001: nonfertile: *b* ± s.e. = 0.005 ± 0.001; *t*_94_ = 4.2, *R* ^2^ = 0.16, *P* < 0.0001). However, the slope of the fertile days was significantly steeper than the one of the nonfertile days (*t*_185_ = 2.12, *P* = 0.035).

**Figure 2:**
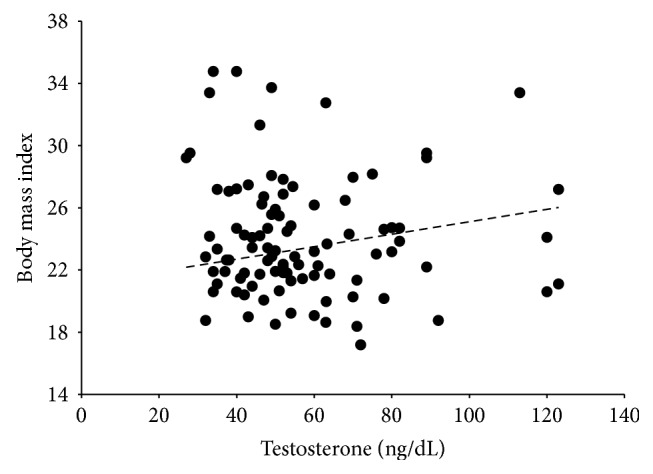
Change in body mass index in relation to salivary testosterone concentrations in women in the nonfertile days of the menstrual cycle. Body mass increased significantly along with testosterone levels (*P* = 0.023).

The BMI was neither correlated with estradiol, testosterone, nor the estradiol × testosterone interaction in women in their fertile days. Yet, in the nonfertile phase, BMI increased significantly as testosterone levels were higher (*b* ± s.e. = 0.04 ± 0.02; *t*_94_ = 2.1, *R* ^2^ = 0.07, adjusted *R* ^2^ = 0.05, *P* = 0.041). Performing the analysis using the raw data yields exactly the same significant effect of testosterone (*b* ± s.e. = 0.04 ± 0.02; *t*_94_ = 2.32, *R* ^2^ = 0.06, adjusted *R* ^2^ = 0.045, *P* = 0.023; Figure 2).

**Figure 3:**
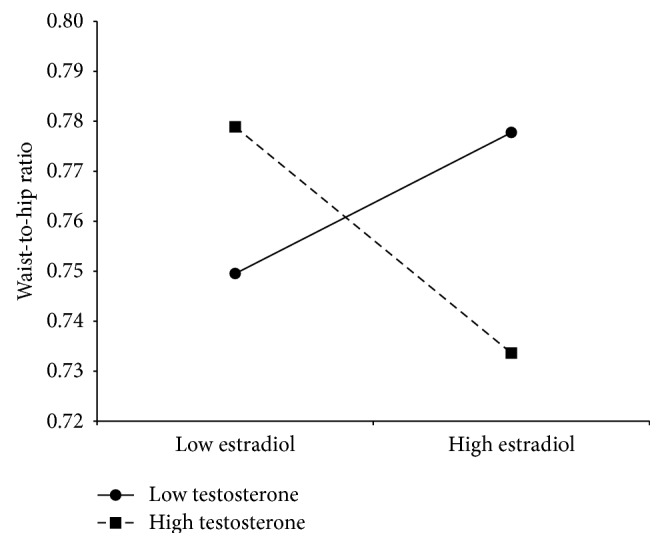
Plot of −1 SD (low) and +1 SD (high) of testosterone and estradiol levels showing the effect on waist-to-hip ratio of the estradiol × testosterone interaction. Low testosterone levels did not have a significant effect on waist-to-hip ratio as estradiol increased (*P* = 0.2). Conversely, when testosterone was high, waist-to-hip ratio changed significantly from being high at low estradiol levels to low when both steroids' concentrations were high (*P* = 0.023).

We found a significant interaction between estradiol and testosterone (*b* ± s.e. = −0.000003 ± 0.000001; *t*_94_ = −2.12, *R* ^2^ = 0.065, adjusted *R* ^2^ = 0.032, *P* = 0.03) in the WHR analyses of the fertile phase.  Figure 3 shows the Aiken and West [49] plot for the interaction between independent variables. Low levels of testosterone had no effect on WHR at varying levels of estradiol (*b* = 0.00007 ± 0.00005; *t*_89_ = 1.4, *P* = 0.2). The high testosterone × estradiol effect showed a significant negative slope (*b* = −0.0001 ± 0.00005; *t*_89_ = −2.31, *P* = 0.023). The highest WHRs were found when testosterone was high and estradiol low, but as estradiol increased, WHR decreased, accounting for the minimal WHRs when both steroids were high (Figure 3). The slopes were significantly different (*t*_180_ = 2.8, *P* = 0.006). No significant effects of estradiol and testosterone on WHR were found in the nonfertile phase.

Finally, we also analyzed the effect of the estradiol/testosterone ratio on BMI and WHR. In neither case we found a significant relationship between the estradiol/testosterone ratio and BMI or WHR controlling for menstrual phase, nor in the total sample (BMI: *b* ± s.e. = −0.073 ± 0.09; *t*_185_ = −0.80, *R* ^2^ = 0.003, *P* = 0.42; WHR: *b* ± s.e. = −0.0 ± 0.001; *t*_185_ = −0.33, *R* ^2^ = 0.001, *P* = 0.74).

## 4. Discussion

Our results show that despite being positively correlated, WHR is not totally dependent on BMI. The coefficient of determination (*R* ^2^) in our study shows that only 21% of the datapoints (39/187) accounted for the significant linear relationship. A look at Figure 1 reveals that the highest WHRs do not account for the most extreme BMIs. Moreover, we found that WHR measures, although slightly, vary between the follicular and luteal phases, while BMI does not, a finding that launches a voice of caution when measuring WHR for either clinical or research studies, since menstrual cycle phase might introduce measurement errors in studies, such as water accumulation throughout the luteal phase.

We found that women having the smallest WHRs during the fertile phase also had the greatest salivary concentrations of testosterone and estradiol. Since testosterone is the precursor of estradiol, our finding could suggest these women might have a higher turnover of estradiol, which, in turn, favors fat deposition in hips and buttocks. However, using the estradiol/testosterone ratio as a proxy of testosterone-to-estradiol conversion did not yield significant results. However, high levels of the nonaromatizable androgen dihydrotestosterone and of sex-hormone binding globulin [35, 50] that inhibit fat accumulation in the abdomen might also contribute to reducing the waist. Our findings show that it is the conjoint action of both estradiol and testosterone (or its metabolites) that results in the “attractive”* ca.* 0.7 WHR [8]. However, more studies are required to understand the metabolic pathways involved.

van Anders and Hampson [34] and Sowers et al. [36] reported a positive correlation between mean bioavailable testosterone levels and female WHR. However, both studies did not measure estradiol. Also, their samples included a large age range, up to 42 years of age, while ours, except for a single case, was restricted to young women going from their eighteens to the late twenties. In women, testosterone decreases monotonically with age, and by 40 women have around half the testosterone concentrations they had by the age of 20 [40]. Therefore, the main difference between the van Anders and the Hampson and the Sowers et al. studies and ours was that our participants were younger; we compared the follicular versus the luteal phase; we measured total estradiol and testosterone levels and included estradiol as a moderator of the testosterone effects. Yet, we cannot attribute our findings to free estradiol or free testosterone effects; therefore, our results are correlative rather than causal. But, considering that our sample encompassed undernourished to obese women (which did not show the highest testosterone levels), we show that high levels of testosterone are not entirely accountable of a high WHR or a high BMI, except in the luteal phase for the latter case. The findings that BMI increased significantly along with increasing testosterone levels in the nonfertile days when estradiol levels are significantly low or that in the fertile days women with the lowest estradiol levels and the highest testosterone levels had the highest WHRs are strong evidence that estrogens levels moderate the testosterone effects. As mentioned above, the metabolic pathways need to be unraveled. Nonetheless, our study shows that testosterone or its metabolites have effects in determining female body shape.

## Figures and Tables

**Table 1:** Descriptive statistics of participants in the fertile and nonfertile phase of the menstrual cycle.

	Fertile					Nonfertile				
	*N*	Mean	Std. deviation	Minimum	Maximum	*N*	Mean	Std. deviation	Minimum	Maximum
Age (years)	91	22.02	2.60	18.00	29.00	96	22.06	2.90	18.00	36.00
Weight (kg)		59.70	10.50	43.00	92.00		61.46	12.46	44.00	107.00
Height (m)		1.58	0.07	1.34	1.80		1.59	0.06	1.48	1.79
Waist-to-hip ratio		0.76	0.05	0.66	0.93		0.78	0.05	0.69	0.93^‡^
Body mass index		23.59	3.52	17.22	34.00		24.04	3.88	17.19	34.77
Estradiol (pg/mL)		233.41	184.77	86.10	1138.10		36.10	25.28	0.00	85.71^***^
Testosterone (ng/dL)		72.02	29.93	20.00	160.00		56.83	21.21	27.00	123.00^***^

^‡^ *P* < 0.1; ^***^ *P* < 0.0001 compared to the fertile phase.
